# High-Precision Aeromagnetic Compensation Method Under the Influence of the Geomagnetic Field

**DOI:** 10.3390/s26061867

**Published:** 2026-03-16

**Authors:** You Li, Guochao Wang, Qi Han, Qiong Li

**Affiliations:** Harbin Institute of Technology, Harbin 150001, China; li.you@hit.edu.cn (Y.L.); 25b303029@stu.hit.edu.cn (G.W.);

**Keywords:** aeromagnetic survey, aeromagnetic compensation, geomagnetic field

## Abstract

Aeromagnetic surveys play an important role in geophysical exploration and many other fields. In many applications, magnetometers are installed aboard an aircraft to survey large areas. Due to its composition, an aircraft has its own magnetic field, which degrades the reliability of the measurements, and thus a technique (named aeromagnetic compensation) that reduces the effect of magnetic interference is required. Commonly, based on a figure-of-merit (FOM) flight, this issue is solved as a linear regression problem. However, the influence of the geomagnetic field, which refers to the magnetic interference introduced by the non-uniform magnetic field in the region, creates accuracy problems when estimating the model coefficients. The analysis in this study indicates that the geomagnetic field can be obtained by a data processing method based on Gaussian-process-regression (GPR) combined with the measurement process. Accordingly, we propose a high-precision compensation method, designated as the Geomagnetic Field-Based (GF-Based) method, which isolates geomagnetic influence to enhance calibration fidelity. This method restricts the impact of the geomagnetic field and improves the precision of the calibration. Compared with the existing methods which considered the geomagnetic field, the proposed method improves the improved ratio (IR), which is verified by a set of airborne experiments.

## 1. Introduction

Aeromagnetic surveys originated in the 1930s for military applications [1] and now play a very important role in many other fields, such as geophysical exploration [2,3]. Magnetic anomaly detection (MAD) detects the relatively weak ferromagnetic target signal hidden in the geomagnetic field by an airborne magnetometer. The acquisition of magnetic data from a plane, helicopter, or drone suffers from static and dynamic magnetic interferences from the platform, and a number of compensation techniques have been proposed to minimize these disturbances (see [4,5] for a list of studies dealing with these techniques, from 1950 to 2021).

The traditional aeromagnetic compensation scheme is based on the attitude information. Commonly, a figure-of-merit (FOM) flight [6] is implemented for calibration, which includes four orthogonal headings and three sets of maneuvers (pitches, rolls, and yaws) in each heading. It requires performing some test flights in an area of nearly constant external magnetic field, a condition that can be approximately achieved at a high altitude [7]. The purpose of choosing high altitudes is to avoid introducing uncontrollable magnetic interference factors from the geomagnetic field into the calibration process. However, certain platforms such as drones or towed sensors often cannot maintain high-altitude flight. Consequently, the magnetometer’s data quality is simultaneously degraded by platform-induced magnetic interference and local geomagnetic field variations [8]. This problem was noticed early on, but until recently, it has not been completely solved.

One way to solve this problem is to distinguish the frequency-domain characteristics of platform magnetic interference and ground magnetic interference, and use frequency-domain filtering to remove them. For example, Ref. [9] attempts to use an adaptive filtering algorithm based on wavelet transform to separate the geomagnetic field’s interference. Groom et al. [10] tried to use high-pass or band-pass filtering to eliminate the influence of low-frequency ground-derived magnetic fields. Qin [11] tried to use the morphological component analysis method to separate the geomagnetic field. A common limitation of these methods is that both filtering and wavelet decomposition are based on the frequency domain, and for the platform magnetic interference and the geomagnetic field with overlapping frequency bands, the separation of geomagnetic field cannot be perfectly solved, but the impact is reduced to a certain extent. Moreover, there is insufficient research on the generalization of frequency-domain methods. The calibration data of different regions and even different flight altitudes are difficult to be separated by a universal filter, which greatly affects the reusability of the method.

In addition, some literature describes the geomagnetic field in terms of fixed coefficients [8] or based on existing geomagnetic field models, such as IGRF geomagnetic models [12,13]. However, much existing data collection occurred prior to the GNSS era, resulting in systematic geographic registration errors that limit the direct application of datasets for describing geomagnetic fields. Recently, some researchers have noticed the influence of the geomagnetic field and proposed a calibration method named Map-Based Tolles–Lawson aeromagnetic compensation [14]. The core idea is to obtain the geomagnetic field through the magnetic map and remove it in the output of the magnetometer, and then use the Tolles–Lawson model to solve it, as shown in Formula (1). However, the details of how to apply the geomagnetic map are not described in detail.

(1) $$\begin{array}{c} H_{scalar}-H_{map}=H_{TL}=A\beta \end{array}$$

With the application of deep learning techniques in aeromagnetic compensation, some studies (e.g., Wang et al., 2025 [15]) have attempted to employ neural networks to directly model and separate aircraft-induced interference from the ambient magnetic field, thereby theoretically avoiding the assumption of ambient magnetic field uniformity. However, such data-driven methods typically rely on extensive training data and substantial computational overhead. In contrast, physics-model-based compensation methods maintain significant importance in practical engineering applications due to their well-defined parametric significance and lower deployment costs.

In the domain of aeromagnetic compensation, the state-of-the-art is represented by the Modified Evolutionary Aeromagnetic Compensation Method [16], which integrates a geomagnetic field model with the classical T-L interference model to simultaneously estimate both variable interference and background field parameters via a recursive least squares algorithm. However, this integrative approach has significant limitations: it provides only a coarse representation of complex geological anomaly fields, and its joint parameter estimation framework fails to decouple the closely intertwined interference sources, leading to potential ambiguity and reduced robustness in complex magnetic environments.

In this work, a high-precision aeromagnetic compensation method named GF-Based (Geomagnetic Field-Based) is proposed. This method focuses on extracting relevant geomagnetic information through a specific acquisition protocol, which complements traditional calibration by improving the characterization of the local magnetic environment. Specifically, we first measure the regional geomagnetic field using geological survey methods and incorporate the difference between the main field and the crustal field into the calibration process. Therefore, the proposed method is more suitable for the separation of the geomagnetic field during calibration.

Compared with existing methods, the GF-Based method achieves calibration results with a higher improvement ratio. The remainder of the paper is as follows. Section 2 presents the mathematical foundations for scalar magnetic field calibration using the Tolles–Lawson method and the geomagnetic field and further introduces the core GF-Based method. Section 3 provides batch calibration results for several calibration methods and presents details on the field experiment. Finally, Section 4 provides conclusions.

## 2. Analysis and Methods

### 2.1. Component Analysis and Model

The measured value $\vec{H_{m}}$ of the magnetic field by a magnetometer in MAD mainly consists of the geomagnetic field $\vec{H_{GF}}$, the airborne maneuvering magnetic interferences in the Tolles–Lawson (TL) model $\vec{H_{TL}}$, and external field disturbances $\vec{H_{EXT}}$ (e.g., diurnal variations and ionospheric and magnetospheric effects). The external field term $\vec{H_{EXT}}$ represents temporal variations such as diurnal changes, which are monitored by a ground base station and subtracted during data preprocessing. For the remainder of this paper, all references to measured magnetic values implicitly assume $\vec{H_{EXT}}$ has been removed, and thus $\vec{H_{m}}$ refers exclusively to the sum of $\vec{H_{GF}}$ and $\vec{H_{TL}}$. Geomagnetic fields can be divided into the Earth’s main field (MF) the crustal field (CF) [17]. So the $\vec{H_{GF}}$ can be divided into the main magnetic field $\vec{H_{MF}}$ and the crustal magnetic field $\vec{H_{CF}}$,

(2) $$\begin{array}{cc} \; & \vec{H_{m}}=\vec{H_{GF}}+\vec{H_{TL}}+\vec{H_{EXT}}\\ \; & \vec{H_{GF}}=\vec{H_{MF}}+\vec{H_{CF}} \end{array}$$

Tolles and Lawson proposed the classical aeromagnetic compensation model to characterize the maneuvering interferences in the 1950s [18]. In the aeromagnetic frame (the x-axis to the left wing, the y-axis aligned with the heading, and the z-axis downward). In Equation (3), $\vec{H_{GF}}$ serves as the inducing field for magnetization effects. The model decomposes the total platform-induced interference $\vec{H_{TL}}$ into three distinct physical contributions based on their magnetic origins: (1) the first term represents permanent magnetization of ferromagnetic components within the aircraft, which is independent of the external field and produces a constant offset proportional to the direction cosines $u_{i}$; (2) the second term represents induced magnetization, arising from the magnetization of soft magnetic materials by the ambient field, which varies with both the field intensity and the orientation; and (3) the third term represents eddy-current magnetization, generated by time-varying magnetic fluxes in conductive structures as the aircraft maneuvers, which depends on both the ambient field and the rate of change of orientation $\dot{\vec{u_{i}}}$. It reads:

(3) $$\begin{array}{c} \vec{H_{TL}}=\sum_{i=1}^{3}a_{i}\vec{u_{i}}+\vec{H_{GF}}\sum_{i=1}^{3}\sum_{j=1}^{3}b_{ij}\vec{u_{i}}\vec{u_{j}}+\vec{H_{GF}}\sum_{i=1}^{3}\sum_{j=1}^{3}c_{ij}\vec{u_{i}}\dot{\vec{u_{j}}} \end{array}$$

where $\dot{\vec{u_{j}}}=d\vec{u_{j}}/dt$. In real applications, the directional vector $\vec{u_{1}},\vec{u_{2}},\vec{u_{3}}$ is measured by a fluxgate magnetometer, which must be rigidly mounted and is usually installed at a magnetically quiet location in the aircraft. These are derived from the three-axis fluxgate magnetometer measurements: the measured vector components(X,Y,andZ) correspond to the projections of the ambient field onto the aircraft body axes, and the direction cosines are computed as:

(4) $$\begin{array}{c} \vec{u_{1}}=X/\sqrt{X^{2}+Y^{2}+Z^{2}}\\ \vec{u_{2}}=Y/\sqrt{X^{2}+Y^{2}+Z^{2}}\\ \vec{u_{3}}=Z/\sqrt{X^{2}+Y^{2}+Z^{2}} \end{array}$$

The TL model is a linear model, so once this disturbance field is calculated, solving for the TL coefficients requires setting up a simple least-squares regression problem of familiar form y=Ax. The process of solving TL coefficients is often referred to as the calibration process, which is based on a dataset from a figure-of-merit (FOM) flight.

The TL model can also be solved using GF-less calibration methods. This is achieved with a clever “trick”—band pass filtering both sides of Equation (2) [19] with a filter that keeps some aircraft fields but removes the geomagnetic field.

(5) $$\begin{array}{c} bpf\left(\vec{H_{m}}-\vec{H_{GF}}\right)=bpf\left(\vec{H_{TL}}\right)\\ bpf\left(\vec{H_{m}}\right)-bpf\left(\vec{H_{GF}}\right)=bpf\left(\vec{H_{TL}}\right) \end{array}$$

Since $bpf(\vec{H_{GF}})\approx 0$, formula (5) can be rewritten in the form of formula (6). At this point, it becomes easier to calculate the TL coefficients.

(6) $$\begin{array}{c} bpf\left(\vec{H_{m}}\right)≅bpf\left(\vec{H_{TL}}\right) \end{array}$$

The band-pass filter is designed to attenuate frequency components outside the typical maneuver-induced interference band (approximately 0.05–0.5 Hz). The underlying physical assumption is that the geomagnetic field $\vec{H_{GF}}$ varies slowly with aircraft position, concentrating its energy at lower frequencies, while platform maneuvering interferences $\vec{H_{TL}}$ occupy a higher frequency band. However, in practice, the filter cannot completely eliminate geomagnetic contributions ($bpf(\vec{H_{GF}})\neq 0$) due to spectral overlap and the aircraft’s finite speed, which translates spatial geomagnetic variations into the time domain within the interference frequency band. Consequently, residual geomagnetic components persist in the filtered signal, necessitating explicit modeling of $\vec{H_{GF}}$ to achieve high-precision compensation.

### 2.2. Representation of the Geomagnetic Field

The GF-less calibration methods do not consider the effect of the geomagnetic field $\vec{H_{GF}}$’s interference and assume that $\vec{H_{MF}}$ or $\vec{H_{CF}}$ is a constant. In Ref. [8], to suppress the geomagnetic gradient interference, also is the $\vec{H_{MF}}$’s interference, the authors used the position information (i.e., longitude $\vec{P_{1}}$, latitude $\vec{P_{2}}$, and altitude $\vec{P_{3}}$) of the airborne platform to build a first-order Taylor polynomial compensation model and added it to the TL model, which can be expressed as follows, where $l_{1}$,$l_{2}$, $l_{3}$ stand for constant coefficients to represent $\vec{H_{MF}}$.

(7) $$\begin{array}{c} \vec{H_{MF}}=\vec{P_{1}}l_{1}+\vec{P_{2}}l_{2}+\vec{P_{3}}l_{3} \end{array}$$

The $\vec{H_{MF}}$ is the strongest, which can reach about 50,000 nT, and the $\vec{H_{CF}}$ is very small, only tens of nT. Therefore, $\vec{H_{MF}}$ and $\vec{H_{CF}}$ need to be considered separately in the process of aeromagnetic calibration.

The $\vec{H_{CF}}$ is modeled as in Equation (8). Because the distribution of the crustal field can be seen as a collection of random variables any finite number of which have (consistent) joint Gaussian distributions, where $\varepsilon ∼N(0,\sigma_{noise}^{2})$ is independent Gaussian noise. Therefore, f(P) can be described by a Gaussian process, where $\vec{P}$ represents a vector of positional elements, and is fully specified by its mean function m(x) and covariance function $k(P,P^{\prime })$. In a non-parametric model, the “parameters” are the function itself.

(8) $$\begin{array}{c} \vec{H_{CF}}=f\left(\left[\vec{P_{1}},\vec{P_{2}},\vec{P_{3}}\right]\right)+\varepsilon \\ f\left(P\right)∼\mathrm{GP}(m\left(P\right),k\left(P,P^{\prime }\right)) \end{array}$$

### 2.3. Model for Crustal Field

The main magnetic field and the crustal field are part of the output of the magnetometer. It has been proved in Refs. [8,20] that the calibration improvement ratio can be further improved by considering the geomagnetic gradient factor and its influence. Ref. [14] proves that the crustal field has a similar effect. Therefore, the GF-Based method in this study focuses on both components of the geomagnetic field, the $\vec{H_{MF}}$ and $\vec{H_{CF}}$.

#### 2.3.1. Analysis

The geomagnetic field can be regarded as as a field with a source. Based on the example of equivalent source simulation, the relationship between the main magnetic field and the crustal magnetic field is given here. Furthermore, we analyze their contribution to the magnetometer output during aeromagnetic calibration.

**Figure 1:**
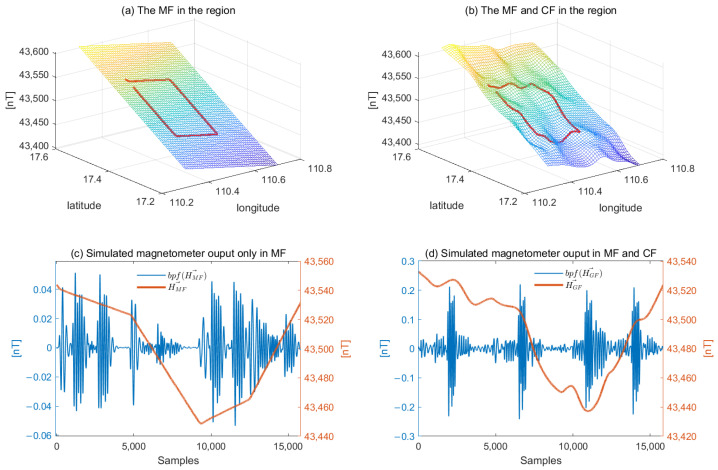
(**a**) The main field within the area is generated with the calibrated magnetic field. (**b**) The main field and crustal field within the area is generated with the calibrated magnetic field. (**c**) A time series representation of the generated calibration data in subfigure (**a**). (**d**) A time series representation of the generated calibration data in subfigure (**b**).

First, an area with a height of 3000 m for aeromagnetic calibration is constructed, and the main magnetic field in the area is obtained by the IGRF model. Several magnetic dipoles are randomly generated in the region. Their positions are randomly distributed, which is used to simulate the crustal magnetic field. As shown in Figure 1a,b, the main magnetic field shows a uniform trend in space, and the magnetic field shows a fluctuating character when the crustal field is superimposed. The red lines represent the introduction of a real flight calibration trajectory under the current geomagnetic field, resulting in a geomagnetic field that does not contain aircraft magnetic interference.

The generated geomagnetic field in the calibration process is represented as a time series, and the curves in Figure 1c,d are obtained, respectively. In Figure 1, it can be seen that the main magnetic field dominates the data component, and the fluctuation observed after filtering (blue line) is caused by the change of the relative position of the aircraft maneuvering action in the geomagnetic field. In Figure 1d, due to the introduction of the geological abnormal magnetic field, the fluctuation in the frequency band is significantly increased and unnecessary noise is introduced. If the noise introduced by the crustal field is not considered, the calibration accuracy will be affected.

**Figure 2:**
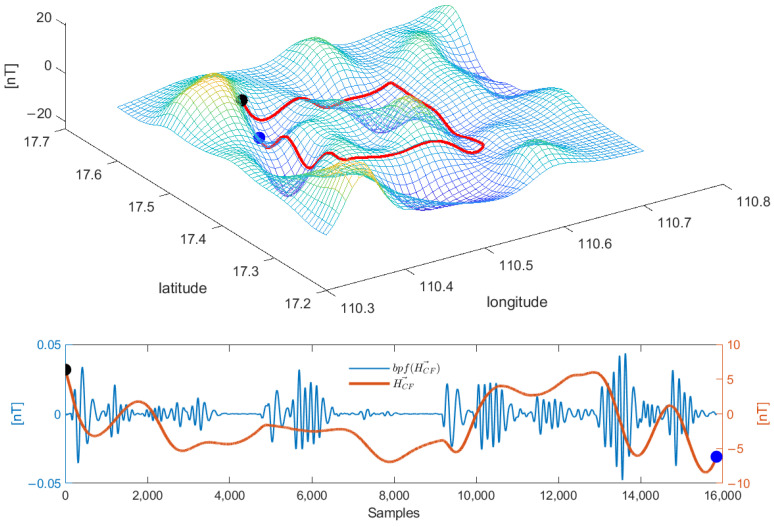
Spatial distribution and time series characteristics of crustal field during calibration.

By stripping the main magnetic field and focusing only on the influence of the crustal field on the calibration process, the simulation results are shown in Figure 2. The original fluctuation range of the crustal field is within 15 nT, but an influence of about 0.1 nT still exists after filtering. Therefore, the existence of the crustal field under high-precision calibration requirements cannot be ignored.

#### 2.3.2. Gaussification of the Crustal Field

Based on the discussion in the previous section, the crustal field may have an impact on the calibration process. In order to describe the crustal field better, it is necessary to determine the model parameters $l_{1},l_{2},l_{3}$ of the main magnetic field first, thereby facilitating the “Gaussification” (transformation to a Gaussian distribution) of the crustal field data. It is important to clarify the role of the IGRF model in this context. The IGRF provides a global representation of the core-derived main field $\vec{H_{MF}}$ with a spatial resolution of approximately 3000 km, corresponding to spherical harmonic degree 13. This long-wavelength model adequately represents the large-scale main field but does not capture local gradients or shorter-wavelength crustal anomalies. In a local survey area spanning only tens of kilometers, the residual magnetic field after IGRF subtraction—denoted $\vec{H_{res}}=\vec{H_{GF}}-\vec{H_{IGRF}}$—contains two components: (1) local main field gradients not resolved by the low-degree IGRF expansion, and (2) crustal anomalies $\vec{H_{CF}}$. Both components vary spatially across the calibration area and contribute to the measured signal during FOM maneuvers.

Our objective is not to modify or “improve” the global IGRF model itself, which would be geophysically unjustified given the spatial scale of our survey. Rather, we aim to model the local residual field $\vec{H_{res}}$ using a first-order Taylor polynomial (Equation (7)) whose coefficients $l_{1},l_{2},l_{3}$ are optimized such that the remaining crustal component after removal approximates a Gaussian distribution. This “Gaussianization” process enables subsequent Gaussian process regression to accurately predict the crustal field within the calibration area. The IGRF thus serves as the initial baseline, and our optimization refines the local representation of the combined main field gradient and crustal anomalies specifically for the purpose of improving aeromagnetic compensation accuracy. This pragmatic approach—prioritizing compensation performance over strict physical interpretability—is consistent with the engineering objectives of this work.

The method of calculating the parameters of the main magnetic field model given in Ref. [8] is not accurate because it ignores the existence of crustal field. However, the method used in Ref. [12] to obtain the main magnetic field by IGRF is limited by the accuracy of the IGRF model, and the gaussification of the crustal field cannot be realized. Therefore, this section presents a method to determine the coefficient of the main magnetic field model through a multi-objective optimization function, and finally realize the gaussification of the crustal field.

The optimal objective uses “kurtosis” and “skewness” to describe the distribution characteristics of crustal field, respectively. The reason for this is to facilitate the Gaussian process regression of the crustal magnetic field after gaussification.

Skewness is a statistic that studies the symmetry of data distribution. By measuring the skewness coefficient, we can determine the asymmetry degree and direction of the data distribution. Kurtosis, is a statistic that studies a steep or smooth distribution of data. By measuring the kurtosis coefficient, we can determine whether the data distribution is more peaked or flatter relative to the normal distribution. Skewness measures the asymmetry of the probability distribution of random variables and is a measure of the degree of asymmetry relative to the mean value. By measuring the skewness coefficient, we can determine the degree and direction of the asymmetry of the data distribution. Specifically, for the random variable *X*, we define skewness SK:

(9) $$\begin{array}{c} SK=\frac{m_{3}}{m_{2}^{3/2}}=\frac{\frac{1}{n}\sum_{i=1}^{n}{\left(x_{i}-\bar{x}\right)}^{3}}{{\left[\frac{1}{n}\sum_{i=1}^{n}{\left(x_{i}-\bar{x}\right)}^{2}\right]}^{3/2}} \end{array}$$

where, $\bar{x}$ is the mean of the samples, $m_{3}$ is the third-order center distance, and $m_{2}$ is the second-order center distance. Skewness is a measure of the asymmetry of the data around the sample mean. If skewness is negative, the data spreads out more to the left of the mean than to the right. If skewness is positive, the data spreads out more to the right. The skewness of the normal distribution (or any perfectly symmetric distribution) is zero.

Kurtosis is a measure of how outlier-prone a distribution is. The kurtosis of the normal distribution is 3. Distributions that are more outlier-prone than the normal distribution have kurtosis greater than 3; distributions that are less outlier-prone have kurtosis less than 3. Some definitions of kurtosis subtract 3 from the computed value, so that the normal distribution has kurtosis of 0. The kurtosis function does use this convention.

The kurtosis KU of a distribution is defined as

(10) $$\begin{array}{c} KU=\frac{m_{4}}{\sigma^{4}}-3=\frac{\frac{1}{n}\sum_{i=1}^{n}{\left(x_{i}-\bar{x}\right)}^{4}}{{\left[\frac{1}{n}\sum_{i=1}^{n}{\left(x_{i}-\bar{x}\right)}^{2}\right]}^{2}}-3 \end{array}$$

In the process of gaussification of the crustal field, it is necessary to optimize the coefficient of the main magnetic field model so that the obtained crustal field is closer to a Gaussian distribution after removing the main magnetic field. Therefore, the optimization target is to achieve KU=0 and SK=0. In this process, the weight coefficients of kurtosis and skewness are set to 1. Here, fgoalattain() is a multi-objective optimization function, and the performance of traditional optimization algorithms is similar, such as max-min method [21] and weighted summation method [22]. The advantages of these traditional algorithms are a solid theoretical foundation, less randomness, and stable performance. Although the main disadvantage is low efficiency, the processing process of the geomagnetic field is offline; therefore, the computational cost of estimating the crustal field using a traditional multi-objective optimization algorithm is acceptable. The steps are described below.

Step 1. Based on the longitude, latitude, and altitude range of the local region and the IGRF magnetic field model, the main magnetic field coefficients $l_{1},l_{2},l_{3}$ in the region are initialized.

Step 2. The initial main magnetic field $\vec{H_{MF}}$ is constructed according to Equation (7). Construct the function $F_{i}$ by Equations (9) and (10),

(11) $$\begin{array}{c} F_{1}=SK\left(\vec{H_{MF}}\right);F_{2}=KU\left(\vec{H_{MF}}\right); \end{array}$$

Step 3. The multi-objective optimization problem is formulated to find the coefficients $l_{1},l_{2},l_{3}$ that minimize the deviation of the skewness $F_{1}$ and kurtosis $F_{2}$ from their target values (zero). This is achieved using the goal attainment method, which solves:

(12) $$\begin{array}{c} \min_{l,\gamma }\gamma \;\mathrm{subject}\;\mathrm{to}\;\frac{F_{i}\left(l\right)-F_{i}^{*}}{w_{i}}\leq \gamma ,\;i=1,2 \end{array}$$

Here, $F_{i}^{*}=0$ are the goal values for skewness and kurtosis (targeting a Gaussian distribution). The weights $w_{i}$ are user-defined positive parameters that determine the relative importance of each objective. The physical significance of the weights $w_{i}$ is that they balance the trade-off between achieving a symmetric distribution (skewness → 0) and achieving a normal tailedness (kurtosis → 0). A smaller weight for a particular objective indicates a higher priority, forcing the solver to bring that objective closer to its goal at the expense of the other. In our implementation, we set $w_{1}=1$ and $w_{2}=1$, assigning equal importance to both statistical moments, as a true Gaussian distribution requires both skewness and excess kurtosis to be zero.

Step 4. The solution process of the fgoalattain function involves transforming the multi-objective problem into a series of single-objective constrained subproblems. The algorithm iteratively adjusts the design variables $l_{1},l_{2},l_{3}$ and the slack variable γ to minimize γ while satisfying the weighted constraints. It employs a sequential quadratic programming (SQP) method, where at each iteration, a quadratic programming subproblem is solved to find a search direction, followed by a line search to ensure progress. The optimization continues until the Karush–Kuhn–Tucker (KKT) conditions are met within a specified tolerance, yielding the optimal coefficients $l_{1},l_{2},l_{3}$ that best achieve the Gaussianization of the crustal field.

(13) $$\begin{array}{c} \left[l_{1}^{\prime },l_{2}^{\prime },l_{3}^{\prime }\right]=fgoalattain\left(\left[F_{1},F_{2}\right],\left[l_{1},l_{2},l_{3}\right],\left[F_{1}^{*},F_{2}^{*}\right],\left[w_{1},w_{2}\right]\right) \end{array}$$

Step 5. The gaussification of the crustal field $\vec{H_{CF}^{\prime }}$ is calculated by using the optimized main magnetic field coefficient.

(14) $$\begin{array}{cc} \vec{H_{MF}^{\prime }} & =\vec{P_{1}}l_{1}^{\prime }+\vec{P_{2}}l_{2}^{\prime }+\vec{P_{3}}l_{3}^{\prime }\\ \vec{H_{CF}^{\prime }} & =\vec{H_{E}}-\vec{H_{MF}^{\prime }} \end{array}$$

The mathematical rationale for this optimization is as follows: The crustal magnetic field $\vec{H_{CF}}$ arises from randomly distributed magnetic sources in the Earth’s crust. According to the central limit theorem, when observed over a sufficiently large spatial scale, the superposition of numerous independent magnetic anomalies tends toward a Gaussian distribution. However, if the main field $\vec{H_{MF}}$ is improperly removed, the residual $\vec{H_{CF}}$ will contain systematic trends that manifest as non-zero skewness and excess kurtosis. By optimizing $l_{1},l_{2},l_{3}$ to minimize |SK| and |KU|, we ensure that the extracted crustal field approximates a zero-mean Gaussian process, which is the fundamental assumption for Gaussian process regression. This “Gaussianization” step is crucial because GPR models rely on the prior that the function f(P) is drawn from a Gaussian process; deviations from Gaussianity would violate this assumption and degrade prediction accuracy. Once the crustal field conforms to a Gaussian distribution, its spatial correlation structure can be effectively captured by the covariance function $k(P,P^{\prime })$, enabling accurate interpolation and prediction within the calibration area.

#### 2.3.3. Prediction of Crustal Field

The Gaussian process regression (GPR) algorithm can be used to establish a regression model between the Gaussianized crustal field and its spatial position. Combined with the position information during the calibration process, the crustal field in the output of the magnetometer can be obtained and then eliminated.

To ensure the reproducibility of the proposed method, we provide a detailed specification of the GPR model configuration. A Gaussian process is fully characterized by its mean function m(P) and covariance function (kernel) $k(P,P^{\prime })$, where $P=[P_{1},P_{2},P_{3}]$ represents the spatial position vector (longitude, latitude, altitude).

Mean Function Selection: We employ a zero mean function, i.e., m(P)=0. This choice is justified because the crustal field data used for GPR has undergone the “Gaussianization” process described in Section 2.3.2, which removes the main magnetic field components and ensures that the residual crustal field has zero mean. The zero mean function simplifies the model without loss of fidelity, as any constant offset would have been eliminated during the main field removal step.

Covariance Function Selection: We select the squared exponential (SE) kernel, also known as the radial basis function (RBF) kernel, as the covariance function. The SE kernel is defined as:

(15) $$\begin{array}{c} k\left(P,P^{\prime }\right)=\sigma_{f}^{2}\exp\left(-\frac{1}{2}\sum_{d=1}^{3}\frac{{\left(P_{d}-P_{d}^{\prime }\right)}^{2}}{l_{d}^{2}}\right)+\sigma_{n}^{2}\delta \left(P,P^{\prime }\right) \end{array}$$

where $\sigma_{f}^{2}$ is the signal variance (controlling the overall magnitude of the function), $l_{d}$ are the characteristic length scales for each spatial dimension (controlling the smoothness and correlation range along longitude, latitude, and altitude), $\sigma_{n}^{2}$ is the noise variance (accounting for measurement noise and residual unmodeled effects), and $\delta (P,P^{\prime })$ is the Kronecker delta function (indicating that noise is independent and identically distributed). The SE kernel is chosen because (1) it provides smooth and infinitely differentiable sample paths, which align with the physical expectation that the crustal magnetic field varies smoothly in space; (2) its automatic relevance determination (ARD) property, enabled by separate length scales $l_{d}$ for each dimension, allows the model to capture different rates of change in different spatial directions; and (3) it is a universal kernel capable of approximating any continuous function given sufficient data, making it well-suited for representing complex crustal magnetic anomalies.

Hyperparameter Optimization: The hyperparameters of the GPR model, denoted as $\theta =[\sigma_{f},l_{1},l_{2},l_{3},\sigma_{n}]$, are optimized by maximizing the log marginal likelihood of the training data. Given a set of training inputs $P_{train}$ and corresponding target values $y_{train}$(the Gaussianized crustal field measurements), the log marginal likelihood is:

(16) $$\begin{array}{c} \log p\left(y_{\mathrm{train}}|P_{\mathrm{train}},\theta \right)=-\frac{1}{2}y_{\mathrm{train}}^{⊤}K^{-1}y_{\mathrm{train}}-\frac{1}{2}\log\left|K\right|-\frac{n}{2}\log2\pi \end{array}$$

where K is the covariance matrix computed from the training inputs using the kernel function, and *n* is the number of training points. This optimization automatically trades off data fit (the first term) and model complexity (the second term), preventing overfitting. We solve this optimization problem using a gradient-based optimizer (specifically, the limited-memory Broyden–Fletcher–Goldfarb–Shanno (L-BFGS) algorithm) with multiple random restarts to avoid poor local maxima. The optimized hyperparameters are then used to make predictions at new positions $P_{*}$ via the standard GPR predictive equations:

(17) $$\begin{array}{c} \mu_{*}=k_{*}^{⊤}K^{-1}y_{\mathrm{train}},\;\sigma_{*}^{2}=k\left(P_{*},P_{*}\right)-k_{*}^{⊤}K^{-1}k_{*} \end{array}$$

where $k_{*}={\left[k\left(P_{*},P_{1}\right),\ldots ,k\left(P_{*},P_{n}\right)\right]}^{⊤}$. This provides both the predicted mean of the crustal field and the associated uncertainty estimate.

The input used to establish the GPR model is still then obtained by the aircraft platform; therefore, the data used for GPR should represent the crustal field in principle but may contain magnetic interference from the aircraft platform. Here, considering that the magnitude and frequency components of the aircraft platform magnetic interference are quite different from the crustal field, the platform magnetic interference components can be treated as part of the noise term in GPR. Therefore, they do not affect the process modeling of Gaussian regression for the crustal field.

**Figure 3:**
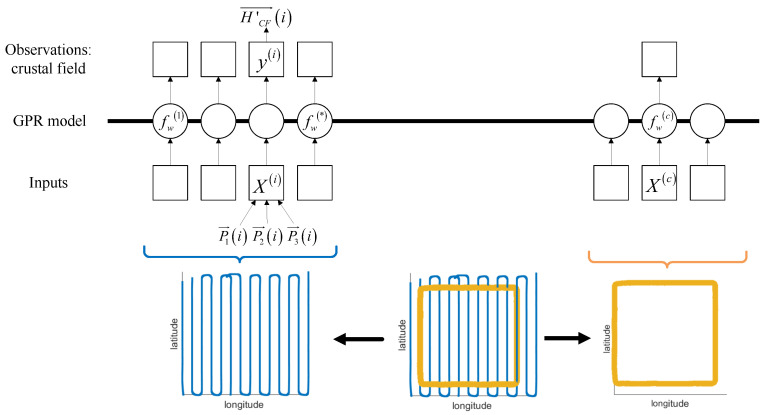
The GPR process is carried out for the crustal field after Gaussification.

The Gaussian process regression of the Gaussification crustal field is shown in Figure 3. In this process, an example is given between the method of obtaining the regional magnetic field and the relative position of the FOM circle. The blue track represents the line-measurement process at the same height as the calibration area, and the yellow track represents the FOM process. The purpose of this design is to obtain an accurate depiction of the crustal field by flying through the same altitude line in a specific area.

**Figure 4:**
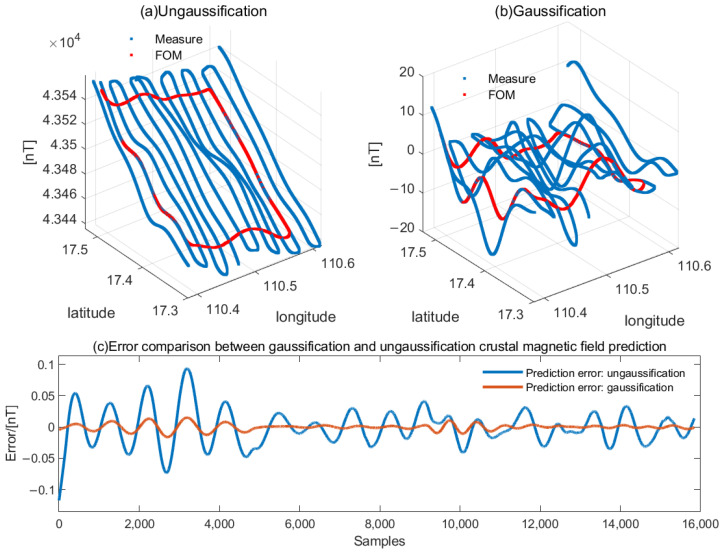
Contribution of Gaussification to crustal field modeling and prediction.

Here, we evaluate the contribution of the Gaussian representation method to the prediction of the crustal magnetic field, utilizing a real flight trajectory along with simulated main and crustal field models. The simulation setup, detailed in Figure 4a,b, illustrates the geometric relationship between the relative position of the measurement line and the designated Figure of Merit (FOM) region. The core procedure involved applying the proposed Gaussification technique to predict the magnetic field within this FOM region based on the available data.

The prediction error resulting from this Gaussified approach is plotted as the red line in Figure 4c. A direct comparison is made against the error profile obtained without employing gaussification, shown as the blue line. The quantitative analysis reveals a significant enhancement in precision: the standard deviation of the prediction error decreases sharply from 0.0284 nT without Gaussification to merely 0.005 nT with it. This substantial reduction in error demonstrates that the Gaussification process effectively captures the underlying spatial structure of the crustal field. Consequently, to achieve a more accurate and reliable description of the crustal magnetic field within the calibration area, the implementation of the Gaussification method is not only beneficial but necessary.

### 2.4. GF-Based Aeromagnetic Compensation Method

**Figure 5:**
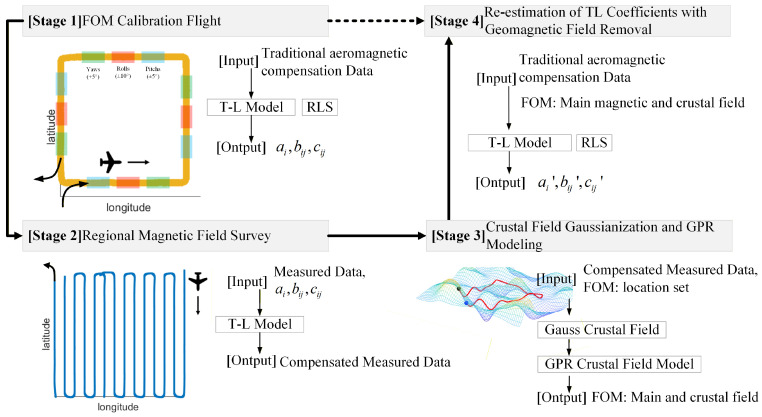
Flow diagram of the proposed GF-Based aeromagnetic compensation method.

Based on the GF-less calibration method, this paper introduces a new measurement process and data processing flow named the GF-Based method to describe and eliminate the crustal field in the process of aeromagnetic calibration. Figure 5 shows the flow diagram of the high-precision aeromagnetic compensation method named the GF-Based aeromagnetic compensation method.

*Stage 1: FOM Calibration Flight*

- **Inputs**: Raw scalar magnetometer data $H_{m}\left(t\right)$, vector magnetometer data (X, Y, Z) for attitude determination, GPS position data (longitude $P_{1}$, latitude $P_{2}$, altitude $P_{3}$).
- **Operational Details**: The aircraft performs Figure of Merit (FOM) calibration maneuvers at a constant altitude of 3000 m (controlled within ±10 m). The maneuvers consist of four orthogonal headings (north, east, south, west), with three sets of maneuvers (pitches, rolls, and yaws) performed in each heading. Each maneuver is executed with an amplitude of approximately $\pm 5^{\circ }∼10^{\circ }$ and a period of 6–10 s to ensure sufficient excitation of the magnetic interference terms.
- **Key Thresholds**: Maneuver quality is monitored in real-time to ensure that the direction cosines $u_{i}$ and their rates of change cover a sufficient dynamic range. A minimum of 10 complete oscillation cycles per maneuver type is required to ensure reliable coefficient estimation.
- **Outputs**: Preliminary Tolles–Lawson coefficients $a_{i},b_{ij},c_{ij}$ estimated using recursive least squares (RLS) algorithm.

*Stage 2: Regional Magnetic Field Survey*

- **Inputs**: Raw scalar magnetometer data $H_{m}\left(t\right)$, vector magnetometer data, GPS position data from survey flights; preliminary TL coefficients from Stage 1.
- **Operational Details**: The same platform conducts survey flights along parallel lines (spaced approximately 2 km apart) at the same altitude as the FOM flight (3000 m ± 10 m). The survey lines are designed to intersect the FOM calibration area, ensuring spatial overlap for accurate geomagnetic field characterization. A ground-based base station magnetometer monitors diurnal variations, which are subtracted from the airborne data during preprocessing.
- **Key Thresholds**: The spatial coverage must include at least 10–15 survey lines crossing the calibration area to provide sufficient training data for subsequent GPR modeling.
- **Outputs**: Platform-compensated magnetic field data $H_{\mathrm{comp}}=H_{m}-H_{\mathrm{TL}}$, where $H_{\mathrm{TL}}$ is computed using the preliminary coefficients.

*Stage 3: Crustal Field Gaussianization and GPR Modeling*

- **Inputs**: Platform-compensated magnetic field data $H_{\mathrm{comp}}$ from Stage 2; GPS position data; IGRF model reference.
- **Operational Details**:
  - **Main Field Optimization**: The coefficients $l_{1},l_{2},l_{3}$ for the main magnetic field model (Equation (7)) are optimized using the multi-objective goal attainment method described in Section 2.3.2. The optimization targets zero skewness and zero excess kurtosis for the residual crustal field, with equal weights $w_{1}=w_{2}=1$.
  - **GPR Hyperparameter Optimization**: The Gaussianized crustal field data is used to train a GPR model with zero mean function and squared exponential covariance function (Equation with ARD). Hyperparameters $\left[\sigma_{f},l_{1},l_{2},l_{3},\sigma_{n}\right]$ are optimized by maximizing the log marginal likelihood using the L-BFGS algorithm with 10 random restarts to avoid local minima.
- **Key Thresholds**: The optimization convergence tolerance is set to $1\times 10^{-6}$ for both the main field optimization and GPR hyperparameter optimization. The GPR model’s predictive performance is validated using 20% of the survey data held out as a test set; the root mean square error (RMSE) on the test set must be below 0.01 nT to accept the model.
- **Outputs**: Optimized main field coefficients $l_{1}^{\prime },l_{2}^{\prime },l_{3}^{\prime }$; trained GPR model f(P) for predicting the crustal field at any spatial position within the calibration area.

*Stage 4: Re-Estimation of TL Coefficients with Geomagnetic Field Removal*

- **Inputs**: Raw FOM flight data from Stage 1 (raw scalar and vector magnetometer data); optimized main field coefficients $l_{1}^{\prime },l_{2}^{\prime },l_{3}^{\prime }$; trained GPR model f(P).
- **Operational Details**: For each time instant during the FOM flight, the main magnetic field $H_{\mathrm{MF}}\left(t\right)$ is computed using the optimized coefficients and the aircraft’s GPS position. The crustal field $H_{\mathrm{CF}}\left(t\right)$ is predicted using the GPR model at the same spatial coordinates. These geomagnetic components are subtracted from the raw scalar magnetometer data:
  $$H_{\mathrm{residual}}\left(t\right)=H_{m}\left(t\right)-H_{\mathrm{MF}}\left(t\right)-H_{\mathrm{CF}}\left(t\right).$$
  The residual signal $H_{\mathrm{residual}}\left(t\right)$ should ideally contain only the platform-induced interference $H_{\mathrm{TL}}\left(t\right)$ and negligible noise. The TL coefficients are then re-estimated using the same RLS algorithm applied to this cleaned dataset.
- **Key Thresholds**: The improvement ratio (IR) of the re-estimated coefficients is computed on a validation dataset. The final coefficients are accepted only if the IR exceeds that of the preliminary coefficients by at least 20%.
- **Outputs**: Final high-precision Tolles–Lawson coefficients $a_{i}^{\prime },b_{ij}^{\prime },c_{ij}^{\prime }$ for operational survey flights.

**Operational Summary**: This enhanced four-stage process ensures that the geomagnetic field (both main field and crustal components) is systematically characterized and removed before final coefficient estimation. The explicit thresholds and validation steps guarantee the robustness and reproducibility of the GF-Based method across different survey areas and flight conditions.

## 3. Field Experiment

The field experiments were conducted in October 2024 in a marine area of southeastern China (centered approximately at 110°30′ E, 17°25′ N). The survey area is characterized by open sea conditions with water depths ranging from approximately 200 to 1000 m. The regional magnetic field in this area exhibits typical oceanic crust magnetic anomalies with moderate spatial variations. No significant anthropogenic magnetic sources (e.g., offshore platforms or pipelines) are present within the survey area.

In order to verify the effectiveness of the GF-Based method, field experiments with a fixed-wing unmanned aerial vehicle (model: CH-4, with a wingspan of 18.0 m and a maximum takeoff weight of 1330 kg) were carried out. In the experiment, to obtain the compensation coefficients, the platform performed roll, pitch, and yaw maneuvers in the directions of east, south, west, and north in a fixed period. FOM altitude and survey altitude were controlled the same height (3000 m). Although most researchers seem to consider that the local geology can be ignored, however, in our experiment, we observed that the geomagnetic field still has influence on the calibration process.

In addition, an optically pumped magnetometer with accuracy <2.5 nT and the range of 20∼100 uT was used for measuring the scalar magnetic field, and its principle can be seen in [23]. A fluxgate vector magnetometer with accuracy <10 nT and a range of −100∼100 uT was used to obtain the motion attitude of the platform. In addition, the Beidou satellite navigation system is used to obtain the position information of the platform in real time. All data were synchronously recorded at a sampling frequency of 10 Hz.

**Figure 6:**
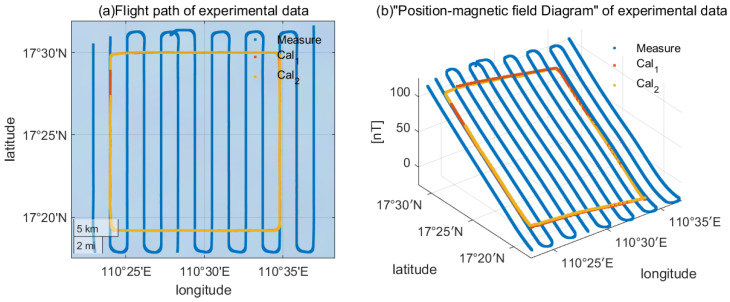
(**a**) Flight path of experimental data. (**b**) Position–magnetic field diagram of the experimental data.

As illustrated in Figure 6a, the experimental design comprised two distinct phases: calibration flights and survey flights. Two FOM calibration flights ($Cal_{1}$ and $Cal_{2}$) were conducted along orthogonal headings to perform calibration maneuvers. These were complemented by 14 north–south oriented survey lines, spaced approximately 2 km apart, to map the regional geomagnetic field. The survey lines were designed to intersect the FOM calibration area, ensuring spatial overlap for accurate geomagnetic field characterization during the calibration process. The relative positions of the 2 FOM flights and measurement lines are shown in Figure 6a.

Regarding the presentation of detected anomalies, we note that the primary focus of this work is the aeromagnetic compensation method itself rather than geological interpretation, while a detailed anomaly map could be generated from the survey data, it would not directly contribute to evaluating the compensation performance. Therefore, we present the raw magnetic measurements in Figure 6b to illustrate the spatial consistency between calibration and survey data, which is the key information for validating the proposed method.

The original measured magnetic field is shown in the position-magnetic field diagram in Figure 6b. In order to observe the variation of the regional magnetic field, data standardization is performed here. The Z-axis in Figure 6b represents the original output of the magnetometer in the MAD which has been standardization. It can be seen that in this region, the spatial consistency between the magnetic field of the measured line and the calibration data is relatively high. In order to avoid the influence of diurnal magnetic field on the deviation of the measurement results for a long time (several hours), the influence of diurnal magnetic field is first removed by monitoring the daily variable magnetic field on ground. Therefore, it can be assumed that the interference other than the platform in the survey line data and calibration data only includes the Earth’s main magnetic field and the crustal magnetic field.

To assist readers in understanding the differences among the comparison methods, we provide a brief summary of their key characteristics. The TLG method [8] extends the classical Tolles–Lawson model by adding first-order position-dependent terms (longitude, latitude, altitude) to account for main field gradients. The TL-IGRF method [13,24] subtracts the IGRF model-predicted main field from the measurements before compensation. The TLG-C method [20] further refines this approach by incorporating both position-dependent terms and main field gradients considerations. The GeoEvo method [16] represents the state-of-the-art, integrating a geomagnetic field model with the TL model, but does not decouple the main field and crustal field components as explicitly as the proposed GF-Based method.

The compensation results of $Cal_{1}$ and $Cal_{2}$ using the compensation models in the literature, TL, TLG [8], TL-IGRF [13,24], TLG-C [20], and GeoEvo [16] are shown in Table 1, together with the GF-Based method. Among them, TLG, TL-IGRF TLG-C and GeoEvo methods all consider the influence of the geomagnetic field. So the GF-Based method is compared with them. To better show the compensation performances of the different methods, Improvement Ratio (IR) [25,26] is used to evaluate their compensation results.

As demonstrated in Table 1, the proposed GF-Based method achieves the highest Improvement Ratios (IRs) across both calibration datasets, outperforming all baseline and state-of-the-art methods. On $Cal_{1}$, our method attains an IR of 10.9776, surpassing the recent GeoEvo method (9.0789) by 20.9% and the conventional TLG-C model (8.7238) by 23.1%. Similarly, on $Cal_{2}$, the GF-Based method achieves an IR of 9.5352, exceeding GeoEvo (8.4103) by 13.4% and TLG-C (7.7111) by 23.1%. These consistent and substantial improvements validate the superior compensation capability of our approach, particularly in handling complex geomagnetic variations.

**Figure 7:**
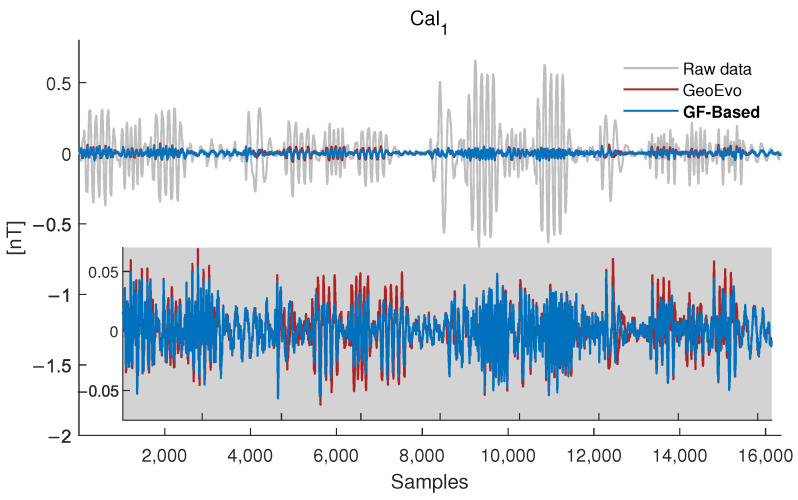
Compensation results of $Cal_{1}$ using the GeoEvo method and proposed method GF-Based.

**Figure 8:**
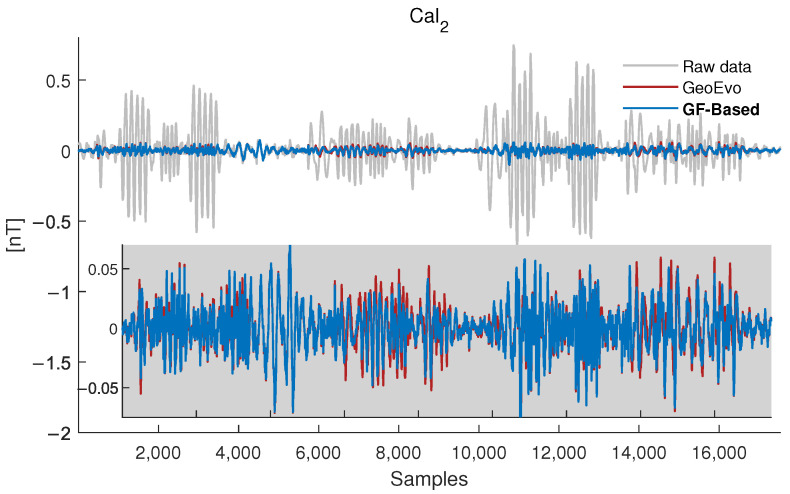
Compensation results of $Cal_{2}$ using the GeoEvo method and proposed method GF-Based.

The compensation results of $Cal_{1}$ using the two compensation methods GeoEvo and GF-Based method are shown in Figure 7. The grey line is the filtered original magnetic field, the red line is the filtered compensation result of the GeoEvo, and the blue line is the filtered compensation result of the proposed GF-Based method. The results show that two methods can both compensate for the maneuver magnetic interference, although the compensation effect of the proposed GF-Based method is better. The compensation results of $Cal_{2}$ are shown in Figure 8, and consistent conclusions can be reached.

The Cross Calibration Index (CCI) [26] is an index for solving robustness (robustness). The robustness of the aeromagnetic compensation method is evaluated by using the ratio of the IR of the results of cross-calibration between different FOM flights. As can be seen in Table 2, the cross-calibration improvement ratio of CCI is close to 1, indicating that the GF-Based method is robust.

Regarding computational efficiency and real-time performance, it should be noted that the GF-Based method requires an additional background survey flight to characterize the regional geomagnetic field, which increases the offline calibration effort. However, this is a one-time cost per survey area. Once the high-precision compensation coefficients are obtained through the GF-Based calibration process, the subsequent real-time magnetic interference compensation during production flights utilizes the same linear TL model as conventional methods. Therefore, the online computational overhead of the GF-Based method is equivalent to that of other approaches, introducing no additional latency or processing burden during actual survey operations.

## 4. Conclusions

In this paper, we take the geomagnetic field, which cannot be ignored during aeromagnetic calibration, as the research object. We propose explicitly compensating for the geomagnetic field during calibration. Moreover, we propose a high-precision aeromagnetic method, named the GF-Based method, combined with a Gaussian process regression algorithm, and provide a prediction method for the crustal magnetic field in the study region. To improve the accuracy of crustal field estimation, we propose a Gaussification algorithm for the crustal magnetic field combined with a description model of the main magnetic field. Field experiments verify that the GF-Based method in this paper achieves higher compensation accuracy than the GF-less method and state-of-the-art methods.

To understand the increasing relevance of geomagnetic field effects, it is essential to situate them within the evolution of aeromagnetic compensation technology. In the nascent stages of this field, when sensor sensitivity was relatively insufficient and compensation residuals remained above the 0.1 nT peak-to-peak threshold, the influence of geomagnetic field variations on coefficient estimation was largely marginal. However, as magnetometer precision has advanced and the demand for high-fidelity geomagnetic data has intensified, traditional compensation methods have reached a performance bottleneck. As residual interference approaches the 0.1 nT level, errors stemming from the neglect of the geomagnetic field—specifically the crustal field component—have transitioned from negligible noise to dominant limiting factors. This shift underscores a critical paradigm requirement: future compensation frameworks must explicitly integrate geomagnetic field variations to surpass current accuracy plateaus.

The principal advantage of the GF-Based method therefore emerges in challenging geological environments where high precision is demanded. The method is particularly beneficial for low-altitude surveys, where geomagnetic field variations are more pronounced due to proximity to crustal magnetic sources. Similarly, in regions with strong geomagnetic anomalies or complex geological structures, the enhanced characterization of both main field and crustal field components yields substantially improved compensation accuracy. The experimental results demonstrate that the improvement ratio advantage of the GF-Based method over conventional techniques increases with the complexity of the background magnetic field, confirming its suitability for demanding applications such as mineral exploration, geological mapping in mountainous terrain, and unmanned aerial vehicle surveys operating at reduced altitudes.

We also acknowledge a limitation of the proposed method: the statistical separation of local main field gradients and crustal anomalies, while physically informed, is not unique and may not perfectly correspond to the true geophysical sources. In regions with highly non-Gaussian crustal fields or complex core field variations, the Gaussianization assumption may be less valid, potentially affecting the compensation accuracy. Future work could explore incorporating independent geophysical constraints (e.g., pre-existing magnetic maps or geological models) to further refine this separation.

Future work will focus on extending the method’s adaptability to diverse geological environments. Potential research directions include: (1) developing adaptive algorithms to automatically adjust the Gaussianization optimization based on regional magnetic characteristics; (2) investigating reduced survey strategies that minimize the additional calibration flight effort while maintaining accuracy; and (3) exploring the integration of real-time geomagnetic field updates using pre-existing magnetic maps or satellite-based models to further enhance operational flexibility.

## Figures and Tables

**Table 1:** Improvement ratio of different methods.

Dataset	TL	TLG	TL-IGRF	TLG-C	GeoEvo	GF-Based
$Cal_{1}$	4.8870	8.0132	8.1089	8.7238	9.0789	10.9776
$Cal_{2}$	4.1675	7.0014	6.9156	7.7111	8.4103	9.5352

**Table 2:** Cross Calibration Index (CCI) of different methods.

Dataset	TLG-C	GeoEvo	GF-Based
$Cal_{1}$	1.0156	1.0203	1.0214
$Cal_{2}$	1.0398	1.0114	1.0211

## Data Availability

No new data were created or analyzed in this study.
